# The effect of exterior traits on milk production and calving ease in Czech Fleckvieh cows in first parity

**DOI:** 10.5194/aab-67-133-2024

**Published:** 2024-04-04

**Authors:** Tomáš Kopec, Radek Filipčík, Blanka Dřízhalová, Pavel Horký, Milan Večeřa, Daniel Falta

**Affiliations:** 1 Department of Animal Breeding, Faculty of AgriSciences, Mendel University in Brno, Zemědělská 1, 613 00 Brno, Czech Republic; 2 Department of Animal Nutrition and Forage Production, Faculty of AgriSciences, Mendel University in Brno, Zemědělská 1, 613 00 Brno, Czech Republic; 3 Department of Animal Husbandry Sciences, Faculty of Agriculture and Technology, University of South Bohemia in České Budějovice, Studentská 1668, 370 05 České Budějovice, Czech Republic

## Abstract

The aim of this study was to analyze the influence of exterior traits on milk production and also on the calving ease in first parity for dual-purpose (milk and meat) cows of Simmental origin. The analysis used 7987 purebred Czech Fleckvieh cows. The impact of the measured features of the frame and the linear type traits of the udder and muscularity were evaluated. The influence of height at the sacrum and body depth on milk yield has been demonstrated. A productivity increase of 27.62 kg of milk can be anticipated for every 1 cm increase in the height at the sacrum, and a productivity increase of 19.78 kg of milk can be expected for every 1 cm increase in body depth. The length of the fore and rear udders, the angle of udder attachment, and the depth of the udders all had a statistically significant impact on milk yield. In the case of calving ease, only the influence of muscularity was proven. The likelihood of difficult calving was 0.18 in cows with weak muscularity. The findings demonstrate that the exterior score is significant not only as a collection of fitness and longevity traits but also as a factor in milk yield.

## 1 Introduction

The Czech Fleckvieh is an important breed of cattle of Simmental origin that is mainly bred in central Europe as a dual-purpose animal under different names (Fleckvieh or Montbéliarde) and is used for the production of milk and beef (Kopec et al., 2013, 2021). The relationship between the exterior and production traits of the Fleckvieh breed has been addressed by a number of authors not only in the Czech Republic (Novotný et al., 2017; Zavadilová et al., 2009) but also in Germany (Ertl et al., 2014; Krogmeier, 2009). However, a sizable portion of existing research on dual-purpose cattle focuses mainly on the connection between exterior traits and longevity (Strapak et al., 2011; Strapáková et al., 2021; Zavadilová et al., 2009).

In Holstein cattle, the relationship between exterior traits, fitness traits, and production traits has also been addressed, such as the connection between measured parameters of the frame and milk production (Konstandoglo et al., 2019). In addition to frame traits, other studies on Holstein cattle have also dealt with udder traits and how they relate to milk production (Khmelnychyi and Karpenko, 2021). Similarly, Foksha et al. (2022) clearly described the problematic relationships between exterior traits and milk yield in Jersey cattle. Furthermore, in Latvia, Petrovska et al. (2017) studied and described the relationship between milk yield and somatic cell count (as dependent variables) and udder exterior parameters (as independent variables) in local dairy breeds. The authors of the latter study pointed, in particular, to a strong correlation between milk production and the udder attachment and height of the udder attachment.

The importance of udder shape in dairy cattle has also been demonstrated by a study (Poppe et al., 2019) that dealt with the use of information from automatic milking systems (AMSs); in the aforementioned work, teat position data obtained from AMSs were used to evaluate the exterior of the udder. For instance, in a study on dairy–meat crossbreeds, the impact of udder size, udder shape, and teat shape on calf gains at weaning was evaluated (Goonewardene et al., 2003). This prior work showed that the importance of a cow's exterior is not only related to its production and fitness traits (Goonewardene et al., 2003).

The influence of the exterior traits on the calving progress was addressed in Holstein cattle in Poland, where the relationship of rump angle and rump dimensions with calving progress was described (Wojcik and Kruk, 2010). Furthermore, the problematics of calving ease in relation to body frame and pelvic dimensions were addressed in a study on Holstein, Brown Swiss, and Jersey cattle populations (Tiezzi et al., 2018). Calving ease is often analyzed in cattle in relation to other important parameters. For example, the mother's nutritional status and body condition score also influence calving ease (Zabransky et al., 2015). Calving ease is mentioned very often in beef breeds of cattle; for example, a study on the Belgian Blue breed looked at the internal dimensions of the pelvis and their influence on the difficulty of calving (Coopman et al., 2003). Moreover, an extensive study in beef cattle dealt with the relationship between reproductive indicators, meat yield, and exterior traits (Berry and Evans, 2014). The authors found that breeding for better reproductive parameters is feasible while also maintaining high demands with respect to production parameters.

The aim of this study was to evaluate the effect of exterior traits on the milk yield and the calving ease in Czech Fleckvieh cows.

## 2 Materials and methods

This study was performed on a set of purebred Czech Fleckvieh dairy cows. The initial database contained 7987 first-parity cows bred in the Czech Republic. The animals were housed on 126 farms under various breeding, diet, herd size, and breeding management conditions.

The dataset included information on calving progress, exterior traits, and milk yield. The data were obtained from the official assessment of Czech Fleckvieh cattle as part of performance monitoring in the Czech Republic. Performance monitoring is carried out in accordance with the ICAR (International Committee for Animal Recording) methodology, and exterior traits are evaluated using the Fleckscore system in accordance with the WSFF (World Simmental Fleckvieh Federation). The exterior of first-parity cows was evaluated between days 30 and 210 of lactation. The selection of individual animals was based on an assessment of the calving progress and the presence of linear type traits. The limiting factor was mainly the number of first-parity cows with an evaluated exterior. In the case of milk productivity and calving progress, a significant part of the population was evaluated. As the majority of the first-parity cows in the dataset came from the test bulls, a random selection from a large enough population can be assumed. On some farms, a comprehensive assessment of first-parity cows in the herd is carried out. First-parity cows of the calving years between 2013 and 2018 were evaluated; however, herds with less than five rated first-parity cows were excluded from the evaluation.

For the purposes of this research, udder traits, muscularity, rump angle, and measured frame traits were selected from the exterior evaluation. Frame parameters were measured by an evaluator using a measuring stick and were expressed in centimeters (cm). The udder traits, muscularity, and rump angle were expressed on a point scale (linear type evaluation of the exterior; see Table 1). The original scale, which ranges from 1 (the lower limit of trait development) to 9 (the upper limit of trait development), was transformed because there were very few point evaluations in the dataset that were close to either biological extreme. Thus, points 1 to 3 and points 7 to 9, in particular, were unified (Table 2). This modified scale was used for testing the influence of the exterior traits on milk productivity and calving ease.

**Table 1:** Description of exterior traits used in the analysis.

Trait	Units	Definition	Point = 1	Point = 9
Height at the sacrum	cm	The measuring point is the imaginary line between the hip bones, and its distance from the ground was measured.	–	–
Rump length	cm	The front measuring point is the beginning of the hip bone. The rear measuring point is the end of the pin bone.	–	–
Body length	cm	The front measuring point is the imaginary extension of the front leg between the shoulder blades. The rear measuring point is the imaginary line between the start of the hip bones.	–	–
Rump width	cm	The measuring point is the outer edges of the hip bones.	–	–
Body depth	cm	The measuring point is the deepest point of the hull without navel.	–	–
Fore-udder length	Points	Description of the length of the fore udder from integration into the abdominal wall to the cross split of the udder.	Extremely short	Extremely long
Rear-udder length	Points	Description of the length of the rear udder from the cross split of the udder seen laterally along a horizontal line to the end of the rear udder.	Extremely short	Extremely long
Fore-udder attachment	Points	Description of the angle between an imaginary vertical line on fore-udder attachment and a line corresponding to the inclination of the fore udder to the transition on the abdominal wall.	Up to 10°, poorattachment	80–90°, excellentattachment
Central ligament	Points	Description of the expression of the central ligament in notching and height. The notch is more important than the height.	Sagging, not noticeableligament	Very noticeableligament and distinctto the top
Udder depth	Points	The distance (in cm) from an imaginary horizontal line in the center of the hock to an imaginary horizontal line at the lowest point of the udder body without teats.	Less than 6 cm	More than 15 cm
Teat placement	Points	Description of the placement of the teats in relation to the udder quarter.	Extremely outwards	Extremely inwards
Teat position	Points	Description of the position of the rearteats.	Ends of the teats point outward from each other	Ends of the teats point towards each other, the teats touch
Teat length	Points	The length of the frontteats (in cm).	Under 2 cm	Over 11 cm
Teat width	Points	The thickness of the frontteats (in cm).	Under 1.50 cm	4.50 cm or more

**Table 1:** Continued.

Trait	Units	Definition	Point = 1	Point = 9
Rear-udder attachment	Points	The height of the hindquarters of the udder. The distance from the lower edge of the vulva to the point where the udder is attached to the body.	More than 40 cm	Less than 20 cm
Muscularity	Points	Description of muscling in the rear upper leg analogous to the SEUROP system (leg muscle convexity).	Very concave,SEUROP = P	Very convex,SEUROP = E
Rump angle	Points	The height difference (in cm) from the top of the hip bone to the top of the pin bone.	Over + 3 cm	Over - 18 cm

Data on milk yields cover the entirety of the first lactation. Because the dairy cows in the dataset had lactation periods that lasted between 240 and 305 d, the effect of the number of days in milk (DIM) was also taken into account in the model when assessing the impact of the exterior traits on milk yield.

The calving ease evaluation was carried out directly by the breeder. A five-point scale was used: a calving ease grade of 1 stands for a spontaneous birth without the assistance of a breeder; a grade of 2 stands for birth with the help of one to two handlers; a grade of 3 is a birth the requires three or more handlers or the help of a veterinarian; grade 4 is assigned for cesarean section or a difficult delivery requiring postpartum treatment with a repeat visit to the veterinarian; and grade 9 indicates missing data or unknown calving progress. Grades 4 and 9 were absent from our dataset. The calving ease (Table 3) was most often spontaneous, and the help of the breeder was not required in most of these first-parity cows (86.91 %). A total of 11.38 % of the first-parity cows received a calving ease grade of 2, and only 1.71 % of these first-parity cows were graded 3. For the purpose of evaluating the influence of the exterior traits on calving ease, the individual grades were modified (Table 3). Due to the extremely low incidence of calving difficulties, this adjustment was required. Grades 2 and 3 were combined into one level (1 on the modified scale), while birth without any assistance was graded 0. This led to the division of births into two categories: (1) spontaneous, unaided birth and (2) birth requiring some kind of assistance from a breeder or veterinarian. Thus, the dependent variable of calving difficulty acquired a binomial frequency distribution.

**Table 2:** Transformation of the original exterior trait scale for the analysis.

Exterior (linear type trait)	
Original	Transformed
scale	scale
1	1–3
2	
3	
4	4
5	5
6	6
7	7–9
8	
9	

### Statistical analysis

The influence of the exterior traits of Czech Fleckvieh dairy cows on the milk yield and the calving ease was analyzed using the statistical program R (R Core Team, 2022). To examine the impact on milk yield, a general linear model with a normal frequency distribution was used. The model included the exterior traits as well as the effects of the herd, the year and season of calving, the age at first calving, and the days in milk (DIM). The effects of age at first calving, days in milk, and all measured exterior traits were tested as second-degree polynomials in the model. On the basis of backward selection, all effects that were nonsignificant according to a Type-I analysis of variance (ANOVA) table ( F test, p<0.05 ) were gradually excluded from the model. The resulting model equation with significant factors, which was used for the interpretation of regression coefficients (Table 6) and testing of means (Table 7) as part of post hoc analysis (Scheffe test, p<0.05 ), took the following form:

$$\begin{array}{rl} y_{ijklmnoprstu} & =\mu +{\text{ farm}}_{j}+{\text{yr}}_{k}+{\text{se}}_{l}+b_{1}{\text{age}}_{i\;}+b_{2}{\text{dim}}_{i}\\ & +b_{3}{\text{hs}}_{i}+b_{4}{\text{bd}}_{i\;}+{\text{ fu}}_{m\;}+{\text{ ru}}_{n}+{\text{fa}}_{o}+{\text{cl}}_{p}\\ & +{\text{ud}}_{r}+{\text{tw}}_{s}+{\text{ra}}_{t}+\mu_{u}+e_{ijklmnoprstu}, \end{array}$$

where y is the dependent variable (kilograms of milk per lactation) for each first-parity cow i ( i=7987 ); μ is the intercept; farm is the herd effect j ( j=126 ); yr is the effect of calving year k ( k=6 , 2013–2018); se is the calving season effect l ( l=4 , December–February, March–May, June–August, September–November); and age, dim, hs, and bd are the respective regressions of age at first calving, day of lactation, height at the sacrum, and body depth for each first-parity cow i with the corresponding regression coefficients $b_{1}$ , $b_{2}$ , $b_{3}$ , and $b_{4}$ . There are also the following exterior effects: fore-udder length (fu, m = 5), rear-udder length (ru, n = 5), fore-udder attachment (fa, o = 5), central ligament (cl, p = 5), udder depth (ud, r = 5), teat width (tw, s = 5), rear-udder attachment (ra, t = 5) and muscularity ( μ , u = 5). e is a random residual error.

**Table 3:** Transformation of the original scale of calving ease for the analysis.

Calving ease –		Calving ease –	
original scale		transformed scale	
Level	N	Level	N
1	6943	0	6943
2	909	1	1044
3	135		

N : number of observations.

A general linear model with a binomial distribution (Bernoulli distribution, GLM-b) was used to assess the impact of exterior traits on the ease of calving. The exterior effects were represented in the model as in the previous model; in addition, the rear-angle effect was tested. The model also took into account the effects of the herd, calving year and season, and milk yield level corrected for DIM. Both the amount of milk produced and the measured parameters of the frame were evaluated as second-degree polynomials. The resulting model equation after removing nonsignificant effects ( χ -square test, p<0.05 , analysis of deviance, ANODEV, Type-I table) was as follows:

$$y_{ijkl}=\mu +{\text{farm}}_{j}+{\text{yr}}_{k}+{\text{mus}}_{l}+e_{ijkl},$$

where y is the dependent variable of calving ease for each first-parity cow i ( i=7987 ), μ is the intercept, farm is the effect of herd j ( j=126 ), yr is the effect of calving year k ( k=6 , 2013–2018), mus is the effect of muscularity l ( l=5 ), and e is a random residual error.

The suitability of the chosen models was assessed on the basis of standard diagnostic diagrams provided by the R program. It was mainly a normal quantile–quantile plot of standardized residuals for the model for milk yield analysis as well as an evaluation of the dependence of the residuals on the predicted values and the standardized residuals, respectively, for both models. No disruptions of the normality of the residuals was found in the first model, and the homogeneity of variances was not fundamentally disturbed in either model (with regard to the binomial character of the second model). For the linear model with a normal distribution, the coefficient of determination (adjusted R -squared value) was 0.49, and the McFadden coefficient of determination estimate for the binomial model was 0.15.

**Table 4:** Descriptive statistics of the primary dataset.

Parameter	Mean	SD	Min	Max
Milk production in first parity (kg)	7152.05	1235.25	2511.00	11 591.00
Age at first calving (d)	819.30	72.56	598.60	1136.80
Days in milk (d)	294.83	14.90	240.00	305.00
Height at the sacrum (cm)	142.06	3.71	124.00	175.00
Body depth (cm)	81.72	2.96	54.00	94.00
Rump length (cm)	53.42	2.14	33.00	63.00
Body length (cm)	87.10	5.23	63.00	106.00
Rump width (cm)	53.69	2.27	41.00	64.00

SD: standard deviation; cm: centimeters; kg: kilograms; Min: minimum; Max: maximum.

## 3 Results

Table 4 describes the default set for analysis. Table 5 lists the factors that had a statistically significant impact on the milk yield. The effects of herd, year, and calving season were highly significant. Age at first calving, day of lactation, height at the sacrum, and body depth were also highly significant covariates. The effects on milk yield of all second-degree polynomials of the relevant covariates that were also tested were statistically inconclusive. Of the measured frame traits, rump length, body length, and rump width were inconclusive. Teat placement, teat position, and teat length were among the measured udder exterior traits, but none of them significantly affected milk production. Other udder traits had a statistically significant effect on milk yield (Table 5), except for the central ligament, where the null hypothesis was rejected at the α=0.05 level. Muscularity also had a highly demonstrable effect on milk productivity. The largest proportion of variability ( F values in Table 5) is explained by the days in lactation (DIM). The fore-udder length, rear-udder length, and height at the sacrum were the most important of the exterior traits. These three exterior traits explained almost 16 % of the variability. Other exterior traits included in the final model had a marginal effect on milk yield variability (around 1 % of total variability).

**Table 5:** Effect of selected factors on milk production.

Factor	Df	F value	Pr ( >F )	Significance
Herd	125	33.2872	$<2.2\times 10^{-16}$	***
Calving year	5	52.8882	$<2.2\times 10^{-16}$	${}^{***}$
Calving season	3	50.6093	$<2.2\times 10^{-16}$	${}^{***}$
Age at first calving	1	129.6094	$<2.2\times 10^{-16}$	${}^{***}$
Days in milk	1	1518.794	$<2.2\times 10^{-16}$	${}^{***}$
Height at the sacrum	1	68.5336	$<2.2\times 10^{-16}$	${}^{***}$
Body depth	1	17.3549	$3.13\times 10^{-5}$	${}^{***}$
Fore-udder length	4	187.8798	$<2.2\times 10^{-16}$	${}^{***}$
Rear-udder length	4	101.4597	$<2.2\times 10^{-16}$	${}^{***}$
Fore-udder attachment	4	23.8722	$<2.2\times 10^{-16}$	${}^{***}$
Central ligament	4	3.1216	$1.4\times 10^{-2}$	${}^{*}$
Udder depth	4	28.7921	$<2.2\times 10^{-16}$	${}^{***}$
Teat width	4	4.6303	$9.8\times 10^{-4}$	${}^{***}$
Rear-udder attachment	4	7.992	$2.00\times 10^{-6}$	${}^{***}$
Muscularity	4	33.9717	$<2.2\times 10^{-16}$	${}^{***}$

Df: degrees of freedom; F value: F statistic from an analysis of variance table; Pr ( >F ): probability of Type-I error based on an F test (rejecting H0 at p<0.05 ); ${}^{***}$ : rejecting H0 at p<0.0001 ; ${}^{*}$ : rejecting H0 at p<0.05 .

Table 6 shows the regression coefficients of the height at the sacrum and body depth on the milk yield obtained from the linear model for milk yield. The height at the sacrum and body depth show a positive relationship with milk yield. A 1 cm increase in the height at the sacrum will result in an extra 27.63 kg of milk produced per lactation. A 1 cm increase in the body depth results in a nearly 20 kg increase in lactational milk productivity.

Table 7 shows the average values of milk productivity for individual levels of exterior traits on a transformed five-point scale. The length of the fore attachment and the rear-udder length show an increase in milk yield with increasing length of the rear and fore udder. Thus, a more voluminous udder is predisposed to a higher milk yield. First-parity cows with a short fore-udder length produced an average of 6191.30 kg of milk per lactation, while those with a significant fore-udder length produced an average of 7594.93 kg. Similar results were found for rear-udder length: cows in grades 1–3 produced an average of 5881.45 kg of milk per lactation, whereas cows in grades 7–9 produced an average of 7584.45 kg of milk per lactation. The angle of fore-udder attachment no longer showed such a clear trend with respect to milk yield. Even cows with a significantly bad fore-udder attachment angle achieved the statistically proven highest milk yield (7275.54 kg of milk per lactation). On the other hand, cows with superior fore-udder attachment had the worst performance. However, the differences in utility between points 4 and 9 are not statistically significant. As expected, first-parity cows with significant central ligament development have the highest productivity (7435.85 kg of milk per lactation), whereas cows with insignificant central ligament development have the lowest productivity (6974.41 kg of milk per lactation). Contrary to the ideal udder formation, with respect to the milk yield in the case of the udder depth trait, first-parity cows with a high-set udder (points 7–9) had a significantly lower milk yield than dairy cows with a low-set udder (below point 5). The average yield of milk per lactation from cows with udder depth scores of 1–3 was 7536.67 kg, whereas the average yield from cows with high udder attachment was only 6842.98 kg. Teat thickness showed a positive trend with milk productivity, i.e., dairy cows with the narrowest teats (points 1–3) had the lowest productivity (7223.53 kg of milk per lactation on average), whereas cows with wide teats produced an average of 7853.26 kg of milk per lactation.

**Table 6:** Regression of the height at the sacrum and body depth with milk production.

Factor	Est. reg. coeff.	SE	t value	Pr ( >|t| )	Significance
Height at the sacrum (cm)	27.6287	3.7717	7.3250	$2.62\times 10^{-13}$	${}^{***}$
Body depth (cm)	19.7699	4.9005	4.0340	$5.53\times 10^{-5}$	${}^{***}$

Est. Reg. Coeff.: estimated regression coefficients; SE: standard error of the mean; t value: t statistic from a t test; Pr ( >|t| ): probability of Type-I error based on a t test (rejecting H0 at p<0.05 ); ${}^{***}$ : rejecting H0 at p<0.0001 .

The height of the rear attachment of the udder, which should ideally be rated at points 7–9, i.e., a high-set udder, also has a positive relationship with milk yield. A dairy cow with a high rear-udder attachment produces an average of 7331.30 kg of milk per lactation; in contrast, a dairy cow with a very low rear-udder attachment produces an average of 6702.07 kg of milk. The muscularity of first-parity cows exhibits an unbalanced trend in relation to milk production. Only first-parity cows with the weakest muscularity (points 1–3) differed from first-parity cows with an average muscularity of 5 in a statistically significant way. First-parity cows with the least amount of muscle produced the most milk on average (7322.94 kg). The milk yield for the other muscularity classes ranged from 7126.86 to 7187.01 kg.

**Table 7:** Effect of exterior traits on milk production.

Factor		LSM		SE	N
Name	Level	Milk yield	Sig.		
Fore-udder length	1–3	6191.30	${}^{e}$	67.59	343
	4	6656.40	${}^{d}$	34.91	1092
	5	6991.51	${}^{c}$	27.15	1916
	6	7233.44	${}^{b}$	23.55	2420
	7–9	7594.93	${}^{a}$	25.38	2216
Rear-udder length	1–3	5881.45	${}^{e}$	94.19	180
	4	6389.83	${}^{d}$	48.42	577
	5	6750.79	${}^{c}$	30.42	1397
	6	7127.85	${}^{b}$	21.58	2832
	7–9	7584.45	${}^{a}$	21.52	3001
Fore-udder attachment	1–3	7275.54	${}^{a}$	24.23	2561
	4	7097.50	${}^{b}$	32.55	1446
	5	7080.56	${}^{b}$	30.31	1733
	6	7145.83	${}^{b}$	31.74	1403
	7–9	7027.95	${}^{b}$	43.05	844
Central ligament	1–3	7071.33	${}^{c}$	28.08	1985
	4	6974.41	${}^{d}$	33.42	1367
	5	7124.01	${}^{\mathrm{bc}}$	29.18	1830
	6	7197.58	${}^{b}$	30.91	1434
	7–9	7435.85	${}^{a}$	32.71	1371
Udder depth	1–3	7536.67	${}^{a}$	97.84	194
	4	7567.49	${}^{a}$	54.49	507
	5	7450.67	${}^{a}$	31.63	1 575
	6	7183.93	${}^{b}$	21.95	2961
	7–9	6842.98	${}^{c}$	22.54	2750
Teat width	1–3	6853.26	${}^{c}$	50.35	558
	4	7085.56	${}^{b}$	28.58	1797
	5	7196.10	${}^{a}$	21.49	3302
	6	7208.70	${}^{a}$	29.69	1739
	7–9	7223.53	${}^{a}$	53.28	591
Rear-udder attachment	1–3	6702.07	${}^{c}$	67.23	288
	4	6862.33	${}^{c}$	43.70	817
	5	7105.24	${}^{b}$	29.22	1922
	6	7179.97	${}^{b}$	22.45	2860
	7–9	7331.30	${}^{a}$	26.38	2100
Muscularity	1–3	7322.94	${}^{a}$	88.96	219
	4	7187.01	${}^{\mathrm{ab}}$	45.03	781
	5	7126.86	${}^{b}$	29.77	1726
	6	7147.84	${}^{\mathrm{ab}}$	21.00	3404
	7–9	7148.33	${}^{\mathrm{ab}}$	28.58	1857

LSM: least-squares mean; Sig.: significance; SE: standard error of the mean; N : number of observations; ${}^{a,b,c,d,e}$ : different superscripts indicate statistically significant differences between the LSM within each factor (rejecting H0 at p<0.05 ).

**Figure 1:**
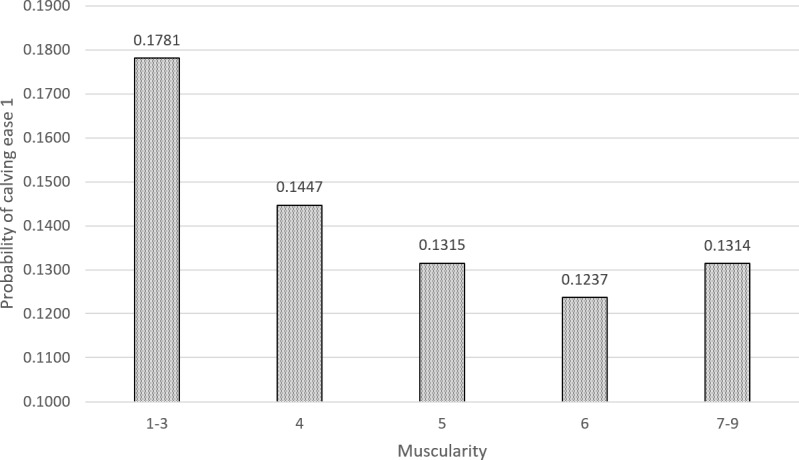
Effect of muscularity on calving ease.

The results of the evaluation of the effect of exterior traits on calving ease are shown in Table 8 and Fig. 1. The model tested the effects of herd, year and season of calving, age at first calving, all measured frame traits, udder exterior traits, and rump angle and muscularity. All effects were statistically inconclusive with the exception of muscularity, calving year, and herd. In the case of herd effect and year of calving, the null hypothesis was rejected at the α=0.001 level; for the muscularity effect, it was rejected at the α=0.05 level. Thus, the influence of the level of milk productivity, the rump angle, and even body dimensions on the calving ease was not proven in Czech Fleckvieh cattle.

**Table 8:** Effect of selected factors on calving ease.

Factor	Df	Deviance	Resid. Df	Resid. Dev.	Pr ( > χ )	Significance
Herd	125	885.79	7861	5307.90	$<2.2\times 10^{-16}$	${}^{***}$
Calving year	5	50.44	7856	5257.50	$1.13\times 10^{-9}$	${}^{***}$
Muscularity	4	11.95	7852	5245.60	$1.7\times 10^{-2}$	${}^{*}$

Df: degrees of freedom; Resid. Df: residual degrees of freedom; Resid. Dev.: residual deviance; Pr ( > χ ): probability of Type-I error based on a χ -square test (rejecting H0 at p<0.05 ); ${}^{***}$ : rejecting H0 at p<0.0001 ; ${}^{*}$ : rejecting H0 at p<0.05 .

The probability of births requiring a certain degree of assistance from the breeder or veterinarian for different levels of muscularity of first-parity cows (grade 1 on the transformed scale) is shown in Fig. 1. The highest probability of the occurrence of problematic calving is in the group of first-parity cows with the weakest muscularity (points 1–3), where the probability reaches a value of 0.18. With an increasing degree of muscularity from point 5 to point 6 in the breeding cows, this probability decreases to values of 0.13 and 0.12, respectively, in breeding cows with excellent muscularity. This means that a higher frequency of difficult calving can be expected in breeding cows with less muscularity.

## 4 Discussion

Our study showed that body depth and height at the sacrum have an impact on milk production. The average height at the sacrum and body depth of Czech Fleckvieh cattle in this work are about 2–3 cm higher than in the most recent thorough study of their exterior from 2017 (Novotný et al., 2017). In the earlier study, a much larger group of breeding cows were evaluated; however, it is important to note that selection pressure on a higher body frame plays a significant role in this breed. In the breeding program of the Czech Fleckvieh, height at the sacrum and body depth are significant indicators, and numerous studies have shown a positive correlation between frame indicators and milk yield (Bardakcioglu et al., 2004; Foksha et al., 2022; Konstandoglo et al., 2019).

A positive relationship between frame and milk yield has also been described in Holstein cattle (Batanov et al., 2021). Additionally, research on 125 Holstein dairy cows demonstrated the beneficial correlation between height at the sacrum and milk production (Bardakcioglu et al., 2011). On the other hand, based on the findings of a study on a population of Brown Swiss and Fleckvieh in Germany, it can be concluded that height at the sacrum is not related to the breeding values of milk production; therefore, selection for a higher body frame does not lead to an increase in milk yield (Krogmeier, 2009). The correlation between animal height and milk yield in Holstein cattle has also been described as being extremely low (Kruszyński et al., 2013). Height at the sacrum has a very high heritability coefficient (Novotný et al., 2017); however, due to the low genetic correlation (Kruszyński et al., 2013) with milk yield, it is difficult to successfully breed for both of these traits simultaneously. A large body frame is considered by breeders to be an important parameter that is a prerequisite for high milk productivity. In general, exterior traits are considered to be significant in relation to production traits (Khmelnychyii et al., 2023). However, many studies (Kruszyński et al., 2013; Strapak and Aumann, 1998) report low (albeit positive) correlation values between milk yield and body frame. For example, the Jersey breed (Lim et al., 2021), which has a small body frame, can have high productivity (after correction for fat and protein content). Another example is Holstein cattle in Israel; these animals are bred for a medium body frame, and the population achieves a high milk yield of over 10 000 kg of milk, as evidenced by some studies (e.g., Weller and Ezra, 2016). Our results support a rather positive relationship between frame and milk yield. In the Fleckvieh breed, a moderately strong correlation between frame and net daily gain is also an interesting fact (Strapak and Aumann, 1998). This is the reason why great emphasis is placed on body frame in the breeding of this dual-purpose breed. This problem with dual-purpose breeds is quite complex, and it is necessary to look at all these characteristics in breeding programs.

For the evaluated udder parameters, the traits associated with udder size showed the strongest correlation with milk yield. This is consistent with research done on the Ongole cattle breed, for which it was found that udder size had a positive impact on milk production (Rao et al., 2021). The importance of udder traits has also been described in other livestock species; for example, udder traits have been reported to be the best predictors of milk yield in goats (Merkhan, 2019). In a population of Holstein cattle kept under conditions typical of the Czech Republic, a statistically significant influence of the central ligament, the length of the fore-udder attachment, the height of the rear-udder attachment, the teat distribution, and the teat length on milk production has been described (Nemocova et al., 2007). Compared with our study, there is a difference in the placement and length of the teats. In our case, these traits do not affect milk production; however, the influence of teat thickness was proven. This may be due to the fact that these breeds differ quite a bit from one another and are of different utility types. A comprehensive analysis of the genetic and phenotypic relationships of all exterior traits and milk production was performed in Holstein cattle (Bohlouli et al., 2015), and the authors of that work describe rather low phenotypic correlations between frame traits and milk yield as well as between udder traits and milk yield. This is consistent with the findings of our research.

An analysis of the relationship of udder exterior and muscularity with milk production was carried out in a small population of mixed Aosta Red Pied cattle (Mazza et al., 2016). The authors of the aforementioned work report a strong positive correlation between milk production and udder traits as well as a moderate negative correlation between muscularity and milk production. The stated relationship between milk production and muscularity corresponds with our results: higher milk yield is observed in cows with worse muscularity. Study of two dual-purpose cattle breeds in Switzerland showed a slightly negative correlation between muscularity and milk production (De Haas et al., 2007). Muscularity is an important indicator of meat production in the Fleckvieh breed, which, in contrast to net gain, also describes the quality of the carcass. Moreover, muscularity has a positive relationship with the body frame (Strapak and Aumann, 1998). Our results, which also point to a positive relationship with calving progress, underline the importance of muscularity. Therefore, great emphasis should also be placed on this parameter during breeding for the Fleckvieh population.

The analysis of the influence of exterior traits on the calving ease only showed a relationship for the trait of muscularity. Research, particularly in beef cattle, has addressed the connection between muscularity and ease of calving. It can be stated that exterior traits do not play a fundamental role in calving ease of Czech Fleckvieh cattle. This is also evidenced by the fact that the probability of difficult calving varied from 12 % to 18 % at different levels of muscularity and was 13 % overall in this work, whereas, for example, the average incidence of births requiring assistance was 31.1 % in a study on Holstein cattle and Holstein crosses with Charolais (Mee et al., 2011). Another study on Holstein cattle reported that 82.10 % of Holstein first-parity cow births are problem-free, which is comparable to our findings. The authors of the aforementioned work report a gradually decreasing incidence of difficult calving in subsequent lactations. Our study only focuses on cows in their first lactation, for which a higher occurrence of difficult calving is expected (Hossein-Zadeh, 2016). Regarding the incidence of difficult calving in dual-purpose cattle, Fleckvieh have been reported to have a difficult calving rate of 15.70 % and Braunvieh have been reported to have a difficult calving rate of 14.29 % (Cziszter et al., 2017). These figures support what we discovered. The influence of the exterior traits on the difficulty of calving is often evaluated in the Belgian Blue breed, for which, for example, the influence of the external and internal dimensions of the pelvis on the occurrence of difficult calving has been proven (Murray et al., 1999). In our results, however, an influence of the exterior dimensions of the pelvis on the frequency of difficult calving was not observed. The impact of the exterior traits on the calving ease has also been dealt with in a study on 900 Holstein dairy cows in Poland, for which an influence of the size of the rump and the rump angle on the incidence of an undesirable course of calving has also been described (Wojcik and Kruk, 2010).

## 5 Conclusions

In conclusion, it can be said that an evaluation of the udder and the frame's exterior traits is crucial, especially for milk production. From the measured frame traits, height at the sacrum and body depth have a positive effect on milk production. The results of the influence of udder traits on the amount of milk are interesting, as traits related to udder size have, as expected, a positive relationship with milk yield. It can also be said that the level of muscularity does not have a clearly negative relationship with the amount of milk produced, which is essential for dual-purpose cattle breeds. It is true that the highest productivity was statistically significantly higher in the most poorly muscled cows; however, the other muscularity classes showed statistically inconclusive differences with respect to milk production between them.

In the case of the influence of the exterior traits on the calving ease, it can be positively assessed that the level of milk productivity had no effect on the calving progress. Additionally, the body frame size and frequently debated rump angle had no detrimental impact on calving ease. The influence of muscularity on calving ease was statistically evident.

## Data Availability

Data are available from the corresponding author upon request.
